# An independent evaluation on the interobserver reliability and intraobserver reproducibility of Toyama classification system for cervical dumbbell tumors

**DOI:** 10.1097/MD.0000000000006183

**Published:** 2017-03-10

**Authors:** Mengchen Yin, Quan Huang, Zhengwang Sun, Xin Gao, Guanghui Chen, Shaohui He, Ye Xia, Junming Ma, Wen Mo, Jianru Xiao

**Affiliations:** a Department of Orthopaedics, LongHua Hospital, Shanghai University of Traditional Chinese Medicine; b Department of Bone Tumor Surgery, Changzheng Hospital, Second Military Medical University, Shanghai, China.

**Keywords:** cervical dumbbell tumor, cervical spine, reliability, reproducibility, Toyama classification agreement study

## Abstract

Dumbbell tumors can not only cause the compression of cervical cord and nerve root, but also invade the important structures and the surrounding organs, causing great harm to the patient. Toyama classification that is commonly used has not been evaluated and still requires independent validation.

The objectives of this study were to evaluate and analyze the interobserver reliability and intraobserver reproducibility of Toyama classification system, explore the differences, discover the shortages, and evaluate the clinical value for diagnosis.

One hundred sixty-five consecutive patients of a cervical dumbbell tumor with complete clinical and radiologic data were enrolled. Six surgeons determined the classification according to Toyama system. The classification was repeated 12 weeks later. Correlation coefficient (ICC) and kappa coefficient (κ) test were used to determine interobserver reliability and intraobserver reproducibility.

The interobserver reliability for Toyama classification was moderate with a value of 0.432. The interobserver reproducibility for Toyama classification was moderate with a value of 0.608.

The Toyama classification has landmark value in clinical practice, but it is a relatively cumbersome system. This study shows that it has low reliability and reproducibility. Accordingly, surgical management of the resection of dumbbell cervical tumors raises several problems, including preservation of the cervical nerve root, control of the vertebral artery, and maintenance of spine. There is a need to optimize the classification in the future.

## 1 Introduction

Dumbbell tumor describes an hourglass-shaped spinal tumor as its growth is hindered by the dura mater, the nerve-root foramen, or other skeletal structures. About 50% of cervical spinal tumors and 15% to 38% of cervical intraspinal canal tumors come up with dumbbell-shaped structures.^[1]^

Tumors such as meningioma, chordoma, chondroma, hemangioblastoma, ganglioneuroma have a dumbbell shape.^[2–5]^ As the most common cervical spine tumor, 70% to 80% of the neuromas are intradural, and 15% to 20% grow along the cervical nerve root inside the foramen,^[6]^ or through the dural aperture, into a dumbbell-shaped extradural or intradural mass.^[7]^ Soft tissues of some neuromas extend under sternocleidomastoid muscle. Some erode or metastasize into vertebral body.

Cervical dumbbell tumors compress the cervical cord and nerve root, and invade the surrounding organs. Due to their varying structures, proximity to the vertebral artery (VA) or carotid sheath, invasion into the vertebral body and different locations, the cervical dumbbell tumors bring great challenges to the surgical management. There is need to formulate a classification system that helps surgeons set up an appropriate and comprehensive strategy. Originating from the classification system of Eden^[8]^ and Hosokawa,^[9]^ the Toyama classification system for cervical dumbbell tumors was first proposed in 2004^[10]^ and has been widely used since then. It has 9 main axially determined categories depending on three-dimensional image information of dumbbell tumors.

This classification system allows surgeons to better understand the treatment of cervical dumbbell tumors. However, it has not been evaluated and still requires independent validation. The goal of this study is to develop a widely accepted comprehensive classification with an independent interobserver and intraobserver reliability.

## 2 Materials and methods

### 2.1 Patient case selection and evaluation

The study was conducted in accordance with the principles of the Declaration of Helsinki. Approval to perform the study was obtained from the ethics committee in our institution. Database records of patients treated in our institution for symptomatic cervical dumbbell tumors between 2006 and 2016 were retrospectively collected and analyzed. To be included by this study, all the patients were required to have complete imaging studies and available clinical data. Patients at presentation and without complete clinical or imaging data were excluded. Complete imaging examination included sagittal view, axial view, and coronal view of magnetic resonance imaging (MRI) to cover all the types and subtypes of the classification system. If bony erosion was suspected, computed tomography (CT) or CT three-dimensional reconstruction was performed. CT angiography (CTA) was introduced when tumors extended into the adjacent major vessels. Complete clinical data included demographic characteristics, chief complaint, neurological function, tumor levels, pathology report, complications, and surgical treatment.

Six surgeons who had collected the cases and treated these patients did not act as evaluator. The evaluators had no information about patients’ identification, original classification, and treatment. To perform a reliable study, each evaluator used the original literature and pertinent information to evaluate the cases according to the classification system. Patients with all types of morphological tumors defined by Toyama classification system were included. Face-to-face meetings and evaluation sessions were added to reach a consensus about the classification. Pertinent information was provided to evaluators along with the complete imaging studies available to avoid image selection bias. And the standard imaging reports were available for evaluators to mimic clinical practice.

Interobserver reliability was determined by comparing the initial responses of all the 6 evaluators. Intraobserver reproducibility was evaluated by comparing 1 evaluator's responses to the same cases, with a 12-week interval and in a random order to limit the recall bias to a minimum.

### 2.2 Statistical analysis

Three statistical tests were used to measure interobserver reliability and intraobserver reproducibility. The intraclass correlation coefficient (ICC) and kappa coefficient (κ) were used to assess both inter- and intraobserver agreement on Toyama classification system (2-way mixed effect model in which people's effects are random and measures’ effects are fixed).^[11]^ The values were expressed with a 95% CI. For each subtype of Toyama classification, Fleiss κ for multiple raters was used to measure interobserver agreement, and Cohen κ was used to evaluate intraobserver agreement.^[12,13]^ Levels of agreement for κ were graded according to the recommendations of Landis and Koch,^[14]^ with κ values of 0.00 to 0.20 considered slightly agreeable, 0.21 to 0.40 as fairly agreeable, 0.41 to 0.60 as moderately agreeable, 0.61 to 0.80 as substantially agreeable, and 0.81 to 1.00 as totally agreeable (Table 1).

**Table 1:**
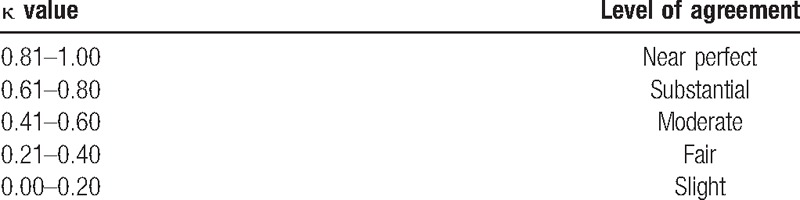
Level of agreement for κ statistic levels.

## 3 Result

The study group involved a total of 165 consecutive patients with a cervical dumbbell tumor who underwent surgeries at our institute between 2006 and 2016. Among these patients were 77 males and 88 females, with a mean age of 47.5 years (range, 7–85 years). All patients underwent X-ray, CT, and MRI before surgery. Tumor locations ranged widely from the upper to the lower cervical spine. The location of the tumor was C1/2 in 43 cases, C2/3 in 24 cases, C3/4 in 25 cases, C4/5 in 29 cases, C5/6 in 19 cases, C6/7 in 20 cases, and C7/T1 in 5 cases. Pathologically, 130 cases were neurinomas, accounting for 79%, 11 ganglioneuromas, 11 neurofibromas, 6 meningiomas, 5 maglignant schwannomas, and 2 primitive neuroectodermal tumors (PNET).

### 3.1 Interobserver reliability

The interobserver reliability of Toyama classification system was moderate with a value of 0.432. The κ value of the 6 physicians was 0.432, 0.563, 0.521, 0.469, 0.498, 0.457, 0.532, 0.521, 0.531, 0.467, 0.567, 0.487, 0.632, 0.646, and 0.456, respectively (Table 2). These values meant moderate agreement.

**Table 2:**
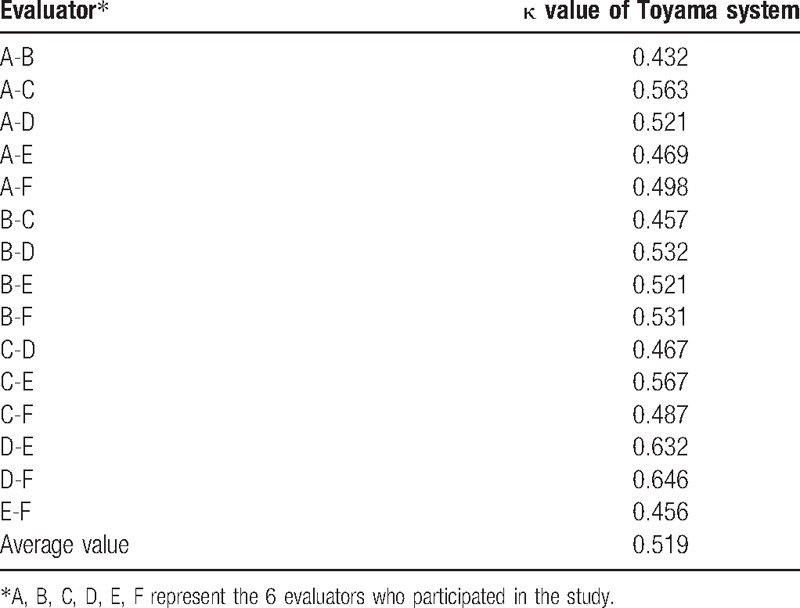
Reliability of Toyama classification system.

### 3.2 Intraobserver reproducibility

The interobserver reliability of Toyama classification was moderate with a value of 0.608. The κ value of the 6 physicians was 0.643, 0.598, 0.675, 0.532, 0.665, and 0.537, respectively (Table 3).

**Table 3:**
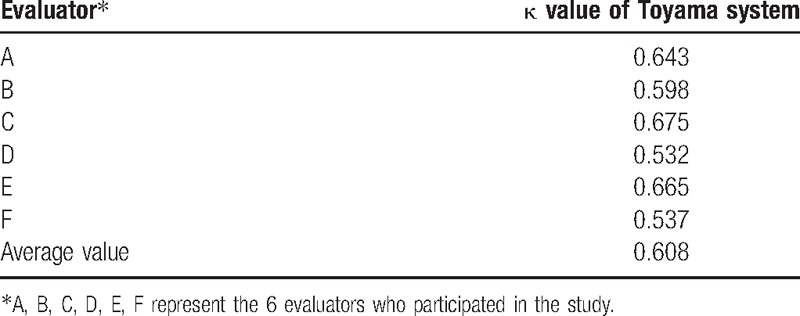
Reproducibility of Toyama classification systems.

## 4 Discussion

Most dumbbell tumors were extramedullary. Radicular pain is the most obvious clinical manifestation, followed by movement and sensory disorders. Dumbbell tumors, especially those in the upper spine, grow in a large space when they extend beyond the intervertebral foramen. So a high rate of misdiagnosis is often made because of insignificant symptoms of spinal cord compression. Advances in computed tomography and magnetic resonance imaging are making the diagnosis of spinal cord tumors easy and precise.

Eden classification system, formulated in 1958 and widely used ever since, is considered a “gold standard” to classify spinal dumbbell tumor.^[8]^ And the classification has been used for a long time. Eden classification designated 4 types of dumbbell tumors are listed without any use of CT and MRI. The difference between tumors and their effect on surrounding nerves, blood vessels, and other tissue structures are not elucidated. So this system is insufficient to guide today's surgery.

Recent years have seen the emergence of some new classification systems. In 2001, Sridhar et al^[15]^ proposed a 5-type classification system for giant invasive spinal schwannomas by incorporating three-dimensional analysis. But this system does not configure out the actions of tumors’ growth extent including and the craniocaudal dimension. Sridhar classification is mainly aimed at schwannomas, and only of type III and type V are categorized as dumbbell-shape tumors. No surgical strategy for different types of tumors was mentioned. So the classification system needs to be improved.^[16]^

**Figure 1:**
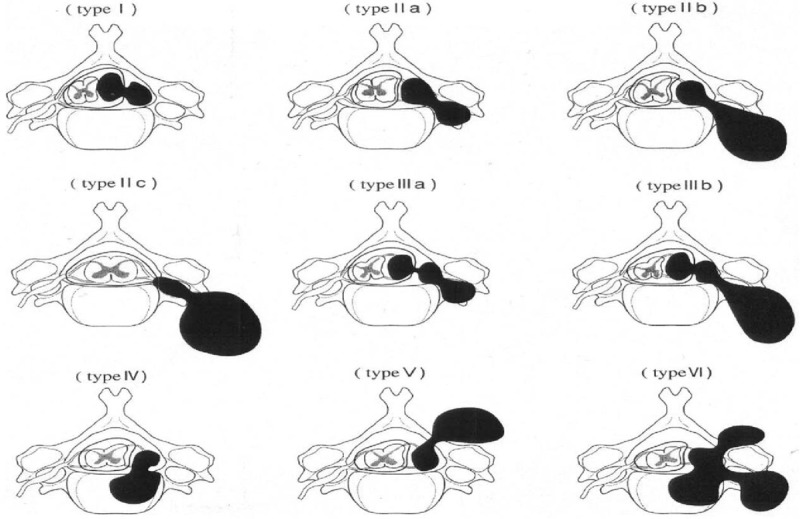
Toyama classification.

With the development of modern imaging techniques, Toyama proposed a new classification system of 9 categories based on three-dimensional CT or MRI information in 2004.^[10]^ Type I: intradural and extradural tumors located in the spinal canal and constricting only the dura; Type II: epidural tumors constricting the foramen, 3 subtypes of a, b, and c defined according to the degree of extra foraminal spread. Type II_a_ tumors do not extend beyond the intervertebral foramen, but types II_b_ and II_c_ do. Thus, type II_a_ is extradural and foraminal; type II_b_ is extradural and paravertebral; type II_c_ is foraminal and paravertebral. Type III: tumors with dural and foraminal constriction. It is divided into 2 subtypes of a and b defined according to positions of tumor and intervertebral foramen. Type III_a_ is intradural and extradural-foraminal; and Type III_b_ is intradural and extradural-paravertebral. Type IV: tumors are extradural and intravertebral, invading only the vertebral body. Type V: tumors are extradural and extralaminal with invasion into the lamina. Type VI tumors show multidirectional erosion of the bone (Fig. 1).

An ideal classification system should be comprehensive, simple, reliable, reproducible, applicable in clinical treatment and prognosis, and communicable for peer review and spreading. According to the literature review, this study is the first to analyze the interobserver reliability and intraobserver reproducibility of the landmark classification systems, compare physicians’ responses to these systems, discover their shortages, and evaluate their clinical value in diagnosis.

Toyama classification demonstrated κ values of 0.432 for interobserver variation and 0.608 for intraobserver variation. The result shows that Toyama classification is not comprehensive and difficult to manipulate. Toyama classification focuses on the anatomical relationship between the tumors and intervertebral foramen or dura. It is easy to categorize and classify most of the tumors, but for some with irregular morphology borders, it is difficult to know whether the tumor is intradural or extradural, beyond or within the intervertebral foramen. For example, Type II_a_ and Type IV, Type II_b_ and Type III_b_ have fuzzy shapes in some cases. MRI also improves the accuracy of the classification, especially the classification of intradural or extradural tumors. A total of 9 categories are too many. In some evaluators’ opinion, too many subtypes do not help clinical practice and research. Besides, Toyama classification system does not include intraspinal lesions in front of the spinal cord. In the case that tumors invade 2 or more lesions, it cannot distinguish them out. In summary, Toyama classification is not comprehensive, reliable, and reproducible.

Toyama classification is helpful in setting up surgical strategy and reconstruction methods. But for an ideal cervical dumbbell tumor surgery, several problems remain unsolved, including how to preserve the nerve root? How to rid the dural defect after tumor removal? How to secure the vertebral artery intraoperatively? How to reduce the recurrence rate and avoid cervical deformity at the same time? How to resect intra- and extradural components? The classification should be optimized in the future work.

The current study has limitations and should be improved to better ascertain the interobserver and intraobserver errors in the classification. Its sample size is relatively small. Increasing the sample size by including nonoperated patients can improve the reliability and reproducibility of these parameters. Another limitation is its retrospective design, and the eventual bias introduced in clinical vignettes, image selection, and recall bias from surgeons. We tried to minimize bias by letting evaluators practice complying with clinical and radiological data. Although recall bias cannot be completely ruled out, we excluded cases in recent 12 weeks to minimize the recall bias. Finally, the clinical experience of physicians was an important factor affecting the accuracy of classification. The long work experience of 6 physicians had insignificant effect for that they were not specialized in cervical tumors. This factor caused different understanding on classification systems and a deviation in the results. Skilled spinal tumor surgeons can be introduced into the study as evaluators to test if they can make an agreement more easily than unskilled surgeons.

In the future, high-quality, large-sample, and multicentered studies should be conducted to provide orthopedic surgeons evidence-based information. To improve the trial quality, future trials should be accomplished with the guidance of CONSORT statement.^[17,18]^

## 5 Conclusion

Although useful in clinical practice, Toyama classification system shows its low reliability and reproducibility in this study. Several procedures should be focused during the resection of dumbbell cervical tumors, including preservation of the cervical nerve root, safeguarding the vertebral artery, and maintenance of the spine. There is a need to optimize the classification in the future.
